# The Other Side of Alzheimer’s Disease: Influence of Metabolic Disorder Features for Novel Diagnostic Biomarkers

**DOI:** 10.3390/jpm10030115

**Published:** 2020-09-06

**Authors:** Chiara Argentati, Ilaria Tortorella, Martina Bazzucchi, Carla Emiliani, Francesco Morena, Sabata Martino

**Affiliations:** Department of Chemistry, Biology and Biotechnologies, University of Perugia, 06123 Perugia, Italy; chiara.argentati89@gmail.com (C.A.); tortorella.i@hotmail.it (I.T.); marti89.b@libero.it (M.B.); carla.emiliani@unipg.it (C.E.); francesco.morena@unipg.it (F.M.)

**Keywords:** amyloid cascade hypothesis, glucose metabolism, adipose tissue dysfunction, energetic metabolism, lysosomes dysfunction, Type-3-Diabetes, neuroinflammation, neurodegeneration

## Abstract

Nowadays, the amyloid cascade hypothesis is the dominant model to explain Alzheimer’s disease (AD) pathogenesis. By this hypothesis, the inherited genetic form of AD is discriminated from the sporadic form of AD (SAD) that accounts for 85–90% of total patients. The cause of SAD is still unclear, but several studies have shed light on the involvement of environmental factors and multiple susceptibility genes, such as Apolipoprotein E and other genetic risk factors, which are key mediators in different metabolic pathways (e.g., glucose metabolism, lipid metabolism, energetic metabolism, and inflammation). Furthermore, growing clinical evidence in AD patients highlighted the presence of affected systemic organs and blood similarly to the brain. Collectively, these findings revise the canonical understating of AD pathogenesis and suggest that AD has metabolic disorder features. This review will focus on AD as a metabolic disorder and highlight the contribution of this novel understanding on the identification of new biomarkers for improving an early AD diagnosis.

## 1. Alzheimer’s Disease: State of the Art

Alzheimer’s disease (AD) is the most widespread neurodegenerative disease and the commonest cause of dementia. Data from The World Health Organization indicate that AD is the fifth cause of death worldwide and predict a dramatic increase in AD incidence in the next years, reaching 152 million people affected by 2050 [1]. This scenario is worsened by the absence of an effective therapy as well as by the absence of an early diagnosis, as it is usually made after symptoms manifestations, when neural impairment and brain injury damages are already severe [2,3,4,5]. One of the major obstacles for the early diagnosis is related to the complexity of AD etiology and pathogenesis [2,3,4,5,6]. It is acknowledged that the amyloid cascade hypothesis, the best model explaining AD development and progression, is not sufficiently clearly described [2,3,4,5,6]. It is also accepted that pathological signs similar to those found in the brain are present in the systemic organs and blood of AD patients [2,3,4,5,7]. 

In this review, we discuss the novel knowledge evidencing AD as a metabolic disorder. In particular, we will describe the contribution to AD pathophysiology of dysfunction in (i) glucose metabolism, (ii) adipose tissue, (iii) mitochondria, (iv) lysosomal compartment functionality, (v) metabolic syndrome, and their correlation with neuroinflammation and neurodegeneration. The influence of these discoveries in the identification of new biomarkers for AD diagnosis is also highlighted.

To be comprehensive, this work starts with the description of the amyloid cascade hypothesis correlating consolidated and new findings.

## 2. The Amyloid Cascade Hypothesis 

The amyloid cascade hypothesis is based on the observation that at least 10–15% of AD patients have an inherited genetic background, while most of the cases (85–90%) are sporadic [4,8]. 

### 2.1. Familial Form of Alzheimer’s Disease

The hereditary form of AD, also known as FAD (familial form of Alzheimer’s disease) usually has an early-onset AD (EOAD), around 30 years of age. FAD is very rare and is caused by dominant inherited mutations in three genes, Amyloid Precursor Protein (APP), Presenilin 1 (PSEN1), and Presenilin 2 (PSEN2), which are implicated in the same metabolic pathway [9,10] (Table 1). APP missense mutations account for 10–15% of EOAD, while more than 272 different missense mutations were found for PSEN1 gene that represent 18% to 50% of the autosomal dominant cases of familial EOAD. PSEN2 gene mutations are the rarest cause of familial EOAD [10,11,12]. The APP gene encodes for the β-amyloid precursor protein, while PSEN1 and PSEN2 encode respectively for presenilin 1 and presenilin 2 subunits of the γ-secretase complex, which are involved in the cleavage of APP to generate the β-amyloid peptide. The alteration of one of these three proteins may steer the physiological non-amyloidogenic pathway to the pathogenic amyloidogenic pathway with consequent accumulation of the amyloid peptide and formation of amyloid plaques, the main hallmark of AD [10,11,12].

### 2.2. Sporadic Form of Alzheimer’s Disease

The sporadic form of Alzheimer’s disease (SAD) has prevalently a late-onset AD (LOAD) on average over 65 years of age [12,14]. SAD represents the majority of the cases of AD and has a more complex pathogenesis than FAD as it may have different potential causes not yet fully understood. During the past decade, clinical and experimental studies have identified many genetic and non-genetic risk factors for SAD [14]. 

Among the genetic risk factors, the presence of mutations on the Apolipoprotein E (APOE) gene correlates with a high probability of developing SAD [14,15]. The APOE gene has three variants, namely ε2, ε3, and ε4 that have different roles in AD pathogenesis. While ε2 and ε3 variants are not involved in AD onset, the ε4 variant is considered the single biggest risk factor for SAD [16]. It has been proposed that APOE-ε4 (APOE4) mediates β-Amyloid (Aβ) aggregation and Tau hyperphosphorylation [3,17]. Moreover, the ApoE protein has immunomodulatory functions [18] by its binding with the microglia Triggering Receptor Expressed on Myeloid Cells 2 (TREM2) receptor [19]. 

Genetic alteration causing Down Syndrome (DS) is another well-established risk factor for AD. As the APP gene is located on chromosome 21, patients with DS that carry an extra copy of this chromosome have a high probability, nearly 90% after 60 years of age, of developing Aβ deposit and AD-related pathological changes [20]. 

Additionally, more than 20 loci associated with SAD have been identified by Genome-Wide Association Studies (GWAS) [21,22]. These genes are involved in different pathways such as glucose metabolism, lipid metabolism, inflammatory pathways, endosomal vesicle recycling, regulation of autophagy, phagocytosis, cholesterol efflux, axon guidance, and cytokine-mediated signaling pathway [2,8,21,23,24,25] (Table 2).

Among the non-genetic risk factors for AD, age, gender, comorbidities, and lifestyle are strictly monitored [27,28,29]. Gender seems to deeply affect the incidence, clinical manifestation, development, and prognosis of the disease, resulting in a higher AD risk for females [30,31,32]. This observation is also confirmed by epidemiologic studies showing that clinical symptoms and neurodegeneration occur more rapidly in women after diagnosis, therefore suggesting that the faster disease progression could be due to neurobiological vulnerability in postmenopausal females [31].

A critical risk is also represented by AD comorbidities such as cerebrovascular diseases (e.g., subcortical leukoencephalopathy, ischemic and hemorrhagic strokes), cardiovascular risk factors (e.g., hypertension, insulin resistance, hyperlipidemia, chronic inflammatory diseases, vitamin D deficiency, alcohol consumption, and smoking) [4] and metabolic alterations (see Section 3). Hypertension may also increase the risk of AD through the alteration of vascular integrity of the Blood–Brain Barrier (BBB), resulting in protein extravasation into brain tissue causing cell damage, reduction in neuronal or synaptic function, apoptosis, increase of Aβ accumulation, and cognitive impairment [33]. 

### 2.3. Molecular Mechanism of β-Amyloid Cleavage

As above mentioned, AD pathogenesis is mostly explained by the mechanisms causing the accumulation of oligomeric β-amyloid-peptide-42 (Aβ42) or related peptides in the brain and their consequent toxic effects on neurons and glia, culminating with programmed neuronal death [34,35].

**Figure 1:**
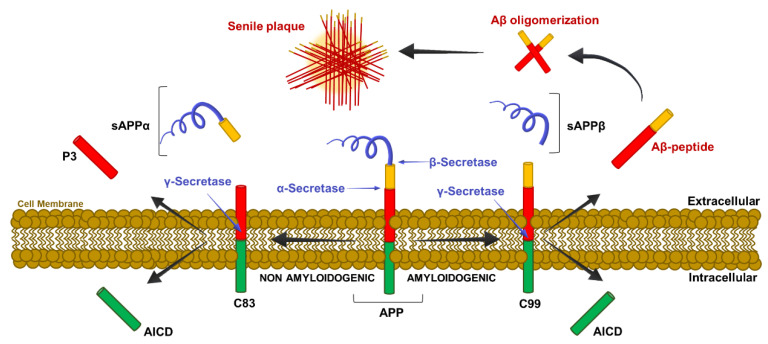
Molecular mechanism of APP cleavage. Cartoon shows the mechanistic events leading the non-amyloidogenic and amyloidogenic process. Details of proteolytic steps are in the text. sAPPα, Soluble Amyloid Precursor Protein α; P3, residues from 17 to 40/42 of the APP; AICD, APP Intracellular Domain; C83, αAPP COOH-terminal fragment 83; APP, Amyloid Precursor Protein; C99, βAPP COOH-terminal fragment 99.

This pathway, called ‘Amyloid Cascade Hypothesis’ is schematized in Figure 1 and is described below. 

The Aβ peptides are the result of incorrect cleavage of the integral membrane protein Type I APP. In the physiological non-amyloidogenic pathway, Type I APP is first cleaved at the *n*-terminal domain by the α-secretase, which triggers the release of Soluble Amyloid Precursor Protein (sAPP) α, which is a large soluble fragment of ectodomain, while the C-terminal domain (the αAPP COOH-terminal fragment (αAPP-CTF), C83) remains in the plasma membrane. Afterwards, the C83 fragment is cleaved by the γ-secretase, generating the soluble non-aggregating and non-neurotoxic peptide P3 (residues from 17 to 40/42 of the APP) that is released in the extracellular space and the APP Intracellular Domain (AICD) fragment, which is released in the intracellular space (Figure 1). 

The toxic amyloidogenic pathway occurs when the β-secretase Beta-site of beta-Amyloid precursor protein Cleaving Enzyme (BACE) cleaves APP at the *n*-terminal domain, releasing the sAPPβ ectodomain and the C-terminal fragments C99 and C100 residues called βAPP COOH-terminal fragments (βAPP-CTFs). After the cleavage by γ-secretase, the Aβ-peptide is released. The γ-secretase may act with two different cuts: the first, at the Valine40, generates the Aβ40 (the most abundant Aβ-peptide isoform), while the second generates the Aβ42 isoform, which accounts for 10% of the total Aβ peptides [36,37,38] (Figure 1).

Aβ is thought to damage neurons directly by increasing the toxicity and promoting hypoglycemia and oxidative stress [5]. Aβ can also act indirectly on neuronal loss by the activation of microglia, leading to the release of toxic and inflammatory mediators such as nitric oxide and cytokines including interleukin (IL)-1β, tumor necrosis factor α (TNFα), and interferon-γ [5,39]. It has also been shown that Aβ oligomers can also induce Tau hyperphosphorylation and, in cultured neurons, neuritic dystrophy [5,39]. 

The presence and the accumulation of Aβ peptides is necessary for the diagnosis of AD, but, as a wide proportion of AD patients die without significant Aβ deposition, it is suggested that this event is not sufficient to completely explain AD pathophysiology [38,40].

#### The Role of Tau Protein 

A corollary of the amyloid cascade hypothesis is the accumulation of Tau Neurofibrillary Tangles (NFTs) [41]. NFTs are intracellular clumps that are mainly composed of paired hyperphosphorylated Tau protein leading to the generation of helical filaments commonly found in neurodegenerative disorders known as “tauopathies” [41,42,43]. 

**Figure 2:**
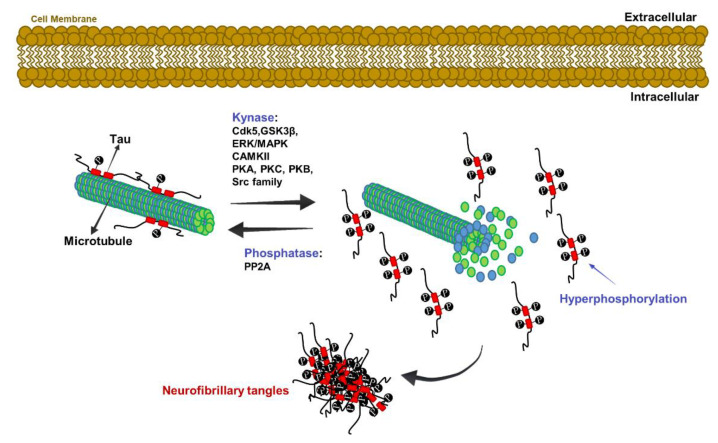
Molecular mechanism of Neurofibrillary Tangles formation. Cartoon shows the mechanistic event leading to altered Tau hyperphosphorylation and the consequent Neurofibrillary Tangles formation as described in the text. CAMKII, Calcium/Calmodulin-dependent Protein Kinase Type II; Cdk5, Cyclin-dependent kinase 5; ERK, Extracellular signal-regulated kinase; GSK3β, Glycogen Synthase Kinase 3 β; MAPK, Mitogen-Activated Protein Kinase; PKA, Protein Kinase A; PKB, Protein Kinase B; PKC, Protein Kinase C; PP2A, Protein Phosphatase 2A; SRC, Proto-oncogene tyrosine-protein kinase Src.

The Tau protein is encoded by the Microtubule-Associated Protein Tau gene located on chromosome 17q21. In normal conditions, it binds microtubules to promote their assembly and stability through a mechanism of phosphorylation and dephosphorylation [44,45] (Figure 2). In AD, Tau hyperphosphorylation causes the loss of its microtubule binding capacity; it also induces the formation of intracellular NFTs and the consequent microtubule depolymerization and defective function [45] (Figure 2). 

After the first report describing and identifying Tau as a phosphoprotein (p-Tau), at least 85 known sites of phosphorylation (mostly serine and threonine, but also tyrosine) are now classified [46,47]. Tau kinases and phosphatase reciprocally balance their activity (Figure 2) [46,47]. For example, Glycogen Synthase Kinase 3 β (GSK3β) activation represses Protein Phosphatase 2A (PP2A), while the repression of PP2A leads to GSK3β activation [48]. Impairment of the Protein kinase B (Akt)/Mammalian Target of Rapamycin (mTOR) pathway may alter the physiological phosphorylation balance between GSK3β and PP2A, as Akt inhibits GSK3β, which in turn inhibits PP2A [45]. Interestingly, it was proposed that the Aβ peptide promotes GSK3β activation, resulting in Tau phosphorylation [49]. Finally, other studies have shown that the acetylation of certain Tau residues (for example, Lys280) can promote Tau autophosphorylation, exacerbate the aggregate formation, and ultimately lead to tauopathy [50].

### 2.4. Biomarkers from Amyloid Cascade Hypothesis for AD Diagnosis

The Food and Drug Administration defines an ideal biomarker as specific, sensitive, predictive, robust, simple, accurate, inexpensive, and measurable in peripheral, easily accessible districts [51,52]. In accordance with the amyloid cascade hypothesis, the National Institute on Aging and Alzheimer’s Association has recognized three categories of biomarkers on the basis of the pathological mechanism in which are involved: Aβ deposits, hyperphosphorylated Tau aggregates, and neurodegeneration [6].

Currently, among the various imaging methods (structural Magnetic Resonance Imaging (MRI), functional MRI, amyloid-Positron Emission Tomography (PET) and 18F-fluorodeoxyglucose (FDG)-PET), amyloid-PET is the most reliable diagnostic tool for AD diagnosis because of its ability to highlight aggregated Aβ peptides within the brain by using amyloid tracers [53]. Similarly, recent new ligands for Tau have been developed such as [18F] PI-2620 and [18F] MK-6240 [54,55,56,57]. Other innovative imaging methods are being developed, including the detection of neuroinflammation process or microglia activation through Translocator Protein-PET [58] and epigenetic modifications and synaptic density/loss [59].

Among fluid biomarkers for AD, the gold standard is the Cerebrospinal Fluid (CSF), because it directly interacts with the brain interstitial fluid and it could reflect pathological changes in AD [60,61]. The typical AD CSF composition is characterized by halved Aβ42-peptide concentration (due to its accumulation in the brain) and an increase of p-Tau and total Tau [60,61,62,63]. In particular, total plasma Aβ42/40-peptide levels were shown to be correlated with amyloid and Tau deposits on a PET scan [64,65].

## 3. Insight into Alzheimer’s Disease as a Metabolic Disorder

As reported above, the current understanding documents the widespread dysfunctions of peripheral organs and blood in AD patients and the contribution of some metabolic alterations to the disease. 

In the following paragraphs, we will discuss in detail the contribution of metabolic alterations associated with AD pathogenesis and the consequent emerged new biomarkers suitable for AD early diagnosis. The section includes (i) aberrant glucose metabolism, (ii) adipose tissue dysfunction, (iii) the alteration of mitochondria, (iv) dysfunction of lysosomal compartment, and (v) metabolic syndrome, and it describes how each of them may improve the identification of new markers for AD.

### 3.1. Glucose Metabolism and AD

**Figure 3:**
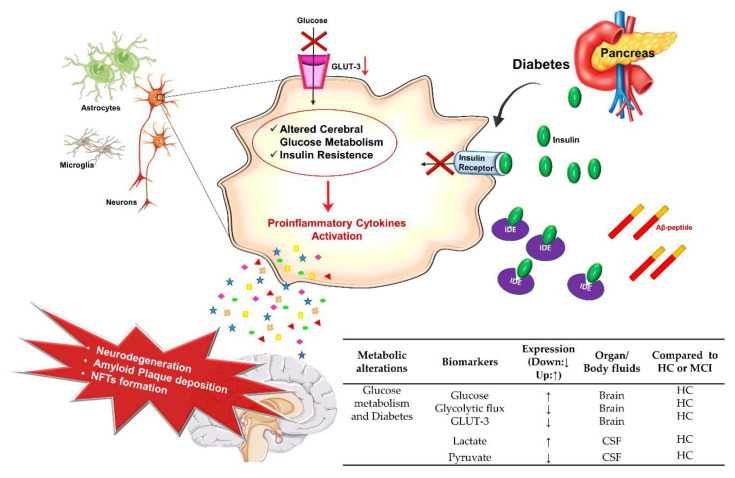
Alteration of glucose metabolism and AD. Cartoon schematizes the main altered events leading to the alteration of glucose homeostasis and reports the table with potentials AD biomarkers. Details are in the text. The arrow direction (up ↑, down ↓) indicates the higher and lower expression levels of the related biomarker in AD with respect to healthy controls (HC). Aβ, β-Amyloid; GLUT-3, Glucose Transporter 3; IDE, Insulin Degrading Enzyme; MCI, Mild Cognitive Impairment; NFTs, Tau Neurofibrillary Tangles.

Dysregulation of the glucose metabolism is a prominent feature in AD patients’ brains [66,67]. Glucose is an essential energy substrate for the maintenance of neuronal activity and is uptaken by glucose transporters expressed in astrocytes, in neurons, and in the cerebral endothelium. A reduction in glucose transporters in both neurons and endothelial cells of the BBB has been documented during AD progression [66] (Figure 3). This includes the glucose transporter 1 (GLUT-1) in glia and endothelial cells and glucose transporter 3 (GLUT-3) in neurons [68]. Experimental evidence showed that GLUT-1 deficiency in endothelial cells in mouse models accelerates AD progression, promoting neurodegeneration, Aβ deposition, and cognitive decline [69].

In a recent study, Yan An and co-authors measured the levels of glucose, GLUT-3, and GLUT-1 in the autopsy brain of AD patients samples that were part of the Baltimore Longitudinal Study of Aging [67,70]. They found a significant higher glucose concentration in the brain of AD patients that correlated with the severity of the disease and symptoms’ onset. Interestingly, they also measured a reduced cerebral glycolytic flux and lower levels of GLUT-3 in the same AD patients [67].

The correlation of altered glucose metabolism in the brain and AD is mostly confirmed by clinical and experimental studies establishing that diabetes, due to insulin resistance, is one of the main risk factors for the development of AD. It is estimated that 65% of diabetic patients have an increased risk of developing AD [71,72,73,74]. 

The role of insulin in brain homeostasis has been investigated in physiology [75,76] and pathology, including AD [77] and diabetes [78]. Insulin is a peptide hormone secreted by pancreatic beta cells that has well-characterized functions in glucose/lipid metabolism and cell growth [79]. It can cross the BBB from the periphery to the Central Nervous System (CNS) competing with the Aβ peptide for the Insulin-Degrading Enzyme (IDE) in the human brain. In the hippocampus, it appears to be involved in the regulation of GSK3β signaling and in the maintenance of neuroplasticity, neurotrophic, and neuroendocrine functions [80]. Altogether, insulin and Insulin-like Growth Factor 1 (IGF1) resistance in AD results in a reduced catabolism of cerebral glucose and promotes oxidative stress, mitochondrial dysfunction, pro-inflammatory cytokines activation, and impaired energy metabolism [81,82,83] (Figure 3). Furthermore, cardiovascular disorders, oxidative stress, inflammation, high level of cholesterol, and Aβ deposition are also common risk factors for AD and diabetes [73].

Collectively, these findings have driven the scientific community to define as ‘type 3 diabetes’ or “brain diabetes” the common molecular and cellular features of type 1 diabetes, type 2 diabetes, and insulin resistance associated with the development of the neurodegeneration [84,85,86,87]. In fact, compared to type 2 and type 1 diabetes, together with insulin resistance or deficiency (canonical hallmarks of diabetes), type 3 diabetes is also characterized by other relevant symptoms such as cognitive decline, impairments in visuospatial function and psychomotor speed flexibility, and loss of attention and memory. Of course, the amyloid aggregation and deposition are also present (see for review [88]).

#### Potential Glucose Metabolism Biomarkers for AD Diagnosis 

Even though today, the role of altered glucose metabolism in AD is well established, the use of related biomarkers is still very moderate because it is difficult to restrict them to AD pathogenesis. Nevertheless, reduced FDG-PET brain metabolism was recognized as a biomarker of neurodegeneration. In fact, FDG-PET detects functions of glucose metabolism, recognizing areas of reduced brain activity and neuronal injury [89,90]. The technique has excellent sensitivity and specificity in discriminating AD from healthy controls [91]. 

In a recent meta-analysis of CSF marker, data highlighted an increased anaerobic glycolysis in AD patients. In particular, it was observed a relevant increase in lactate and a decrease in pyruvate, whereas the levels of glucose and glutamate in the CSF of AD patients were comparable to control subjects [92] (Figure 3). Conversely, as mentioned above, the glucose level increased in the brain of AD patients, while the glycolytic flux and GLUT-3 levels decreased.

### 3.2. Adipose Tissue Dysfunction and AD

Several studies have associated the excess of body weight with an increased risk of AD [82,93,94,95]. Therapies for reinstating metabolic homeostasis could improve cognitive functions in AD patients [96,97,98], while a high-fat diet might exacerbate cognitive function in animal models of AD [99,100,101]. 

**Figure 4:**
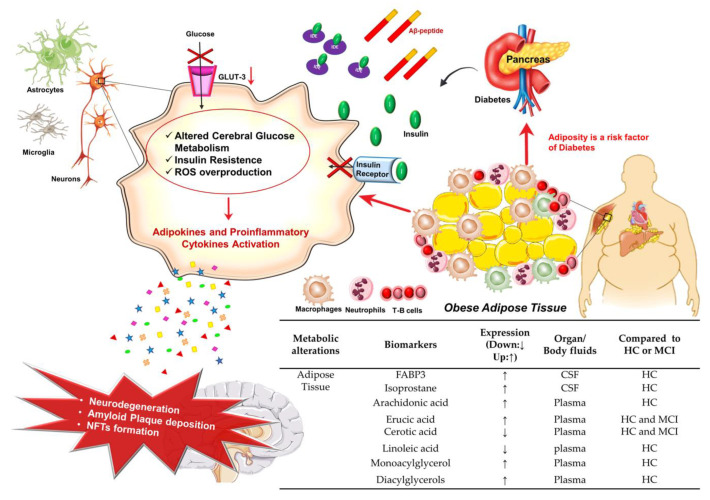
Adipose tissue dysfunction and AD. Cartoon schematizes the main altered events leading to the alteration of adipose tissue homeostasis and reports the table with potentials AD biomarkers. Details are in the text. The arrow direction (up ↑, down ↓) indicates the higher and lower expression levels of the related biomarker in AD with respect to healthy controls (HC) or MCI (Mild Cognitive Impairment). Aβ, β-Amyloid; FABP3, Fatty Acid-Binding Protein; GLUT-3, Glucose Transporter 3; IDE, Insulin Degrading Enzyme; NFTs, Tau Neurofibrillary Tangles.

There is a number of potential mechanisms linking high adiposity to AD (Figure 4). For example, the increase of free fatty acids, which is common in obesity, contributes to the onset of insulin resistance [102]. In addition, adiposity is a risk factor for diabetes, hypertension, and cardiovascular changes, all conditions contributing themselves to a significantly increased risk of AD [93,103].

The adipose tissue plays a central role in regulating the body energy and the homeostasis of glucose, both at organs and systemic levels [104,105,106,107,108]. In particular, adipose white tissue stores energy in the form of lipids and controls the mobilization and distribution of lipids in the body, while adipose brown tissue maintains body temperature and acts as an endocrine organ, producing numerous bioactive factors such as adipokines (e.g., Leptin, Adiponectin, Apelin, Resistin, Monocyte Chemoattractant Protein-1 (MCP-1), IL-1β, IL-6, IL-10, TNFα, Transforming Growth Factor β (TGF-β)) and lipokines (such as Lysophosphatidic Acid, LPA), which control many metabolic pathways including in the brain [104,105,106,109,110]. In particular, Leptin has pro-inflammatory activities, maintains neurogenesis and synaptogenesis, and is involved both in neuroprotection and neuroinflammation. Adiponectin controls the proliferation of hippocampal neural stem cells as well as the release of somatotropins and gonadotropins, and it partakes in the neurodegeneration process. Resistin is necessary for cognitive function performance and hypothalamic insulin resistance. LPA is critical for brain development, neurogenesis, the proliferation and differentiation of neural progenitor cells, and synaptic transmission. TNFα controls neurogenesis, neuroprotection, and the survival of neural progenitor cells [111]. The role of adipokines/lipokines able to cross the BBB and enter in the CNS is further confirmed by the fact that their misexpression might alter or disrupt directly/indirectly brain’s homeostasis and functions [105,106,109]. As a matter of fact, alterations of the BBB structure such as in inflammatory conditions cause an increased permeability to adipokines and LPA into the CNS and an increase of oxidative stress and neurodegeneration [111]. Interestingly, adipokines can also activate endothelial cell receptors, resulting in the modulation of tight junctions expression and BBB permeability [111]. Finally, adipokine dysregulation and oxidative stress were also involved in the remodeling of blood vessels and arterial stiffness in high-fat diet mice [112]. Altogether, these findings suggest that in pathological conditions, adipokines promote Reactive Oxygen Species (ROS) overproduction and inflammatory processes, which are involved in BBB disruption and could potentially act on different brain regions such as the hippocampus (Figure 4). This could explain why metabolic dysfunctions are associated with hippocampus atrophy and with an increased risk of developing dementia and AD [113]. In this regard, systemic alterations have been correlated to chronic inflammation caused by adiposity [114,115,116].

#### Potential Adipose Tissue Biomarkers for AD Diagnosis

Considering the important role that adipose tissue plays in AD pathogenesis, several studies were carried out to monitor lipids and lipid metabolism-related molecules in the peripheral districts of AD and Mild Cognitive Impairment (MCI) patients (Figure 4). For example, it has been observed a higher CSF level of Fatty Acid-Binding Protein 3 (FABP3) compared to healthy subjects [117,118] (Figure 4). Moreover, higher CSF levels of FABP3 and isoprostane were observed in MCI patients that evolved toward AD [117,118,119,120]. Different types of lipids such as arachidonic acid, erucic acid, monoacylglycerol, and diacylglycerol were shown to be higher in AD compared to MCI and healthy subjects, whereas other lipids were found to be lower, including cerotic acid and linoleic acid [119,120,121,122,123,124,125] (Figure 4).

In the last years, several studies aimed to understand if alterations in adipokines expression could be used as a biomarker for AD progression. Current data are contradictory, perhaps due to the intricate interplay in which these biomolecules are involved [126,127,128,129,130,131,132,133,134,135,136]. Thus, the measurement of adiponectin levels in CSF or plasma led to divergent results: reduced levels of adiponectin have been observed in the serum [137] and CSF [138] of AD patients, while in other studies, plasma and CSF adiponectin were significantly higher in MCI and AD compared to controls [139]. Wennberg et al. found that serum adiponectin is positively correlated to amyloid levels but inversely correlated to the hippocampal volume in women with MCI [140].

### 3.3. Energetic Metabolism, Mitochondria Dysfunction, and AD

**Figure 5:**
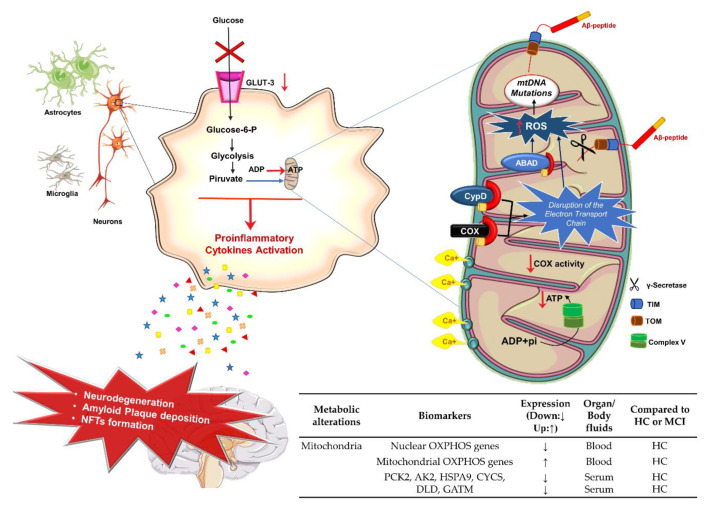
Energetic metabolism, mitochondria dysfunction, and AD. Cartoon schematizes the main altered events leading to the alteration of energetic metabolism and mitochondria dysfunction in AD described in the text and the table reports the potential AD biomarkers. The arrow direction (up ↑, down ↓) indicates the higher and lower expression levels of the related biomarker in AD with respect to healthy controls (HC). ABAD, Aβ Binding Alcohol Dehydrogenase; ADP, Adenosine Diphosphate; AK2, Adenylate Kinase 2; ATP, Adenosine Triphosphate; Aβ, β-Amyloid; COX, Cytochrome C Oxidase; CypD, Cyclophilin D; CYCS, Cytochrome C; DLD, Dihydrolipoyl dehydrogenase; GATM, Glycine Amidinotransferase; Glucose-6-P, Glucose 6-Phosphate; GLUT-3, Glucose Transporter 3; HSPA9, Stress-70 protein; MCI, Mild Cognitive Impairment; mtDNA, mitochondrial DNA; NFTs, Tau Neurofibrillary Tangles; OXPHOS: Oxidative Phosphorylation; PCK2, Phosphoenolpyruvate Carboxykinase [GTP]; Pi, Inorganic Phosphate; ROS, Reactive Oxygen Species; TIM, Translocase of the Inner Membrane; TOM, Translocase of the Outer Membrane.

Over the years, age-related alterations of mitochondria functioning have been observed in different neurodegenerative diseases such as AD [141,142,143]. An impairment of cellular bioenergetics has been observed in AD patients [144,145], and mitochondrial dysfunction appears to be an early event in AD pathogenesis [146,147] that is deeply correlated to the initiation of neuroinflammation [148]. Different observations have been made in an attempt to explain the mechanisms behind mitochondrial dysfunctions observed in AD (Figure 5). 

First, neuronal mitochondria in AD are different in number and shape. The mitochondria number per neuron is reduced as the physiological balance between fission and fusion is altered, leading to a decreased biogenesis [149], and they appear to be swollen with misshapen cristae [150]. It has been observed in the AD brain of transgenic mice that there is a physical association of Aβ peptide and mitochondria [151] occurring through the involvement of the translocase of the outer membrane machinery [152]. It has also been shown that the Aβ peptide interacts directly with intracellular proteins such as the mitochondrial enzyme Aβ Binding Alcohol Dehydrogenase (ABAD), thus mediating mitochondrial enzymes insufficiency, oxidative stress, and cell death [153,154] (Figure 5). 

Even though the exact mechanism of how Aβ peptide damages neuronal cells is still unknown, several studies elucidated that the interaction of Aβ peptide with mitochondrial proteins, such as Cyclophilin D (CypD) [155] and Cytochrome C Oxidase (COX) [156], leads to a disruption of the Electron Transport Chain with a consequent increase in ROS production and loss of Adenosine Triphosphate (ATP) generation [157]. Interestingly, COX activity was reported to be reduced also in platelet mitochondria derived from AD patients and in animal models of AD [158,159] (Figure 5). Moreover, the Aβ peptide appears to interfere with antioxidant enzymes (i.e., superoxide dismutase [Cu-Zn], catalase, and glutathione) and Kreb’s cycle enzymes (pyruvate dehydrogenase, malate dehydrogenase, and aconitase) [160]. On the other hand, other reports suggested that the mitochondrial dysfunction is independent of Aβ-peptide deposition and hypothesized the ‘Mitochondrial cascade hypothesis’ for AD pathogenesis, by which the impairment of mitochondrial activity (mostly due to the respiratory chain and bioenergetic mechanisms failures) is upstream the Aβ cascade formation [161,162].

Mitochondria normal homeostasis appears to be compromised in the AD brain at different levels, including Ca^2+^ homeostasis, which regulates various neuronal functions such as impulse transmission, synaptic plasticity, and neuronal death. It has been observed that Aβ/Tau mitochondrial binding impairs also the mitochondrial Ca^2+^ handling capacity, resulting in oxidative stress, decreased mitochondrial membrane potential, decreased mitochondrial permeability transition pores formation, and deficient ATP synthesis [163]. On the one hand, impaired intraneuronal Ca^2+^ signaling is known to promote the abnormal aggregation of Aβ peptide, while on the other hand, Aβ can lead to a cytosolic Ca^2+^ overload by reducing its storage in the endoplasmic reticulum [144].

As a result of its proximity to the principal intracellular source of ROS and the lack of protective histone and limited repair mechanisms, mitochondrial DNA (mtDNA) is particularly susceptible to DNA damage caused by oxidative stress. As a matter of fact, several studies observed that AD-mtDNA differs from age-matched control subjects. Brain mtDNA nucleotide oxidative damage, as well as the frequency of point mutations, appear to be higher in AD subjects [164,165]. Moreover, association studies highlighted that particular mtDNA haplogroups and specific mtDNA SNPs are statistically over or underrepresented in AD patients [166,167,168,169].

Interestingly, recent studies have demonstrated a correlation between APOE4 expression and the mitochondrial function [170,171]. In particular, Schmukler and co-author have demonstrated that mitochondria have reduced fission and mitophagy in APOE4-carrier astrocytes compared to APOE3-carrier astrocytes [170]. These results were also confirmed in the hippocampus of ApoE4 mice [171].

#### Potential Mitochondria Biomarkers for AD Diagnosis 

Considering the central role of mitochondria in AD pathophysiology, a major issue for AD early diagnosis is the identification of new candidate biomarkers related to mitochondrial enzymes and metabolism in peripheral districts such as CSF and blood. Even though the establishment of reliable and consistent mitochondrial biomarkers for AD is still not achieved, studies have been carried out to shed light on the possible use of mitochondrial markers for this purpose [172]. For example, a recent study has demonstrated the possibility of detecting alterations in the expression of nuclear and mitochondrial encoded Oxidative Phosphorylation (OXPHOS) genes in blood samples from AD and MCI patients [173] (Figure 5). Interestingly, a recent serum proteome profiling study identified deregulated proteins in AD samples, in particular, 12 proteins associated with mitochondrial function including PCK2, AK2, HSPA9, CYCS, DLD, and GATM (see Abbreviation List) [174] (Figure 5). In another study aiming at identifying differentially expressed genes in blood samples of AD patients, it has been suggested that mitochondrial dysfunction, nuclear factor-κB (NF-κB) signaling and inducible Nitric Oxide Synthase (iNOS) signaling pathways are all dysregulated in AD pathophysiology [175]. However, these results still need to be confirmed. Finally, giving the importance of mitochondria in AD pathogenesis, Ridge et al. recently created a new extended dataset of mitochondrial genomes to investigate the impact of mitochondrial genetic variation on the risk for AD, with the aim of helping further research on mitochondrial peripheral biomarkers [176].

### 3.4. Lysosomes Dysfunction and AD

In post-mitotic neurons, endocytosis and macroautophagy processes are particularly important for maintaining the correct homeostasis and transducing signals through axons and synapses; thus, the correct functioning of the lysosomal system is crucial for the nervous system [177,178,179]. 

Many reports have associated autophagic pathway dysfunction to neurodegeneration development [180]. Alterations of this pathway have been identified at different levels and resulted in defective autophagic proteolysis of the macromolecules/molecular complexes within the lysosomal compartment [180,181,182]. The correlation of abnormal autophagic activity in AD has been described by Nixon and co-authors [180], who emphasized the impairment of the autophagolysosomes in human AD fibroblasts and in several models of the disease [183,184]. For instance, they have demonstrated the role of presenilin-1 for targeting v-ATPase to lysosomes as well as for their acidification and autophagic activity. These functions were absent in cells from psen1-null mice [184]. The abnormal autophagosomes accumulation was also recently described in the distal region of axons in neurons of AD patients and animal models, and it was correlated with the impairment of retrograde transport along the axons and with changes in the autophagic clearance of the Aβ peptide [179]. This work is an example of many others showing the autophagy deficit in the “neuronal housekeeper” function by which lysosomes lose the capability to degrade aberrant proteins, thereby participating in the accumulation Aβ peptide and Tau [185,186,187,188,189,190,191,192,193].

Interestingly, the enlargement of early endosomes/lysosomes compartments in neurons of AD brains as well as the extracellular presence of lysosomal hydrolases and their co-localization with amyloid plaques have been also observed before amyloid deposition [194,195,196,197]. During AD progression and in primary tauopathies, lysosomes become compromised, as there is an accumulation of endo/autolysosomal structures and intermediates in dystrophic neurons around amyloid plaques [197,198,199,200]. Many other laboratories have reported marked lysosomal system alterations in mouse models of AD (both amyloidosis and tauopathy models [201,202,203]). which had been noted also in brain regions affected only at very late stages of the disease [204,205], thereby suggesting that lysosomal alterations could precede neurodegeneration and encouraging further studies focused on the initial stages of AD [206]. Moreover, lysosomal impairment seems to be accompanied by the activation of particular secretory routes to remove misfolded protein, including Tau and Aβ peptide [207,208,209,210], as observed in Neuroaminidase 1 (Neu1) null mutant mice [211]. This could diminish the intracellular proteins accumulation but facilitate altered cell-to-cell transmission of the pathology.

Overall, the connection between AD and lysosomal dysfunction appears to be a vicious cycle in which lysosomal impairment contributes to Aβ peptide and Tau accumulation which, in turn, contributes to improper functioning of the lysosomal pathways [212].

In particular, Aβ peptide aggregates were found in endosomes, autophagic vesicles, multivesicular bodies, and lysosome [196,213,214,215], where the presence of APP in the outer lysosomal membrane and γ-secretase activity in lysosomal membranes was shown [216,217,218]. In addition to being a potential site of Aβ-peptide production, lysosomes are responsible for the complete hydrolysis of APP, APP-CTFs, and Aβ [219,220]. A recent study in APP/*psen1* mice highlighted the importance of lysosomal degradation of APP in preventing its availability to the canonical amyloidogenic pathway [220]. Moreover, over-activation of the lysosomal system is emphasized in EOAD caused by mutations of *psen1* and in transgenic mice overexpressing the PS L146M mutation [221]. Lysosomal system activation progressively worsens as neurons become metabolically compromised. Loss of function gene mutations of Cathepsin D (CatD), a ubiquitous lysosomal protease [222], have been shown to cause progressive neurodegeneration [206,223,224,225,226] and, additionally, CatD was found to be decreased in AD patients’ fibroblasts [227] and mouse models [228]. Cathepsin B (CatB) was also found to be decreased in a mouse model [228], and the evidence of its important role in reducing Aβ-peptide levels reinforced the interest in cathepsins role in AD [229]. Further evidence of lysosomal dysfunction in AD came from alterations in lysosomal enzymes expression and activity. In particular, activities of the lysosomal glycohydrolases β-Galactosidase (Gal), β-Hexosaminidase (Hex), and α-Mannosidase were found to be increased in fibroblasts from AD patients and presymptomatic individuals with FAD [230] and in the cortex of mouse model of AD, where there was an increase in Gal and Hex activity [231]. Studies conducted in our laboratory led to the observation that alterations in lysosomal glycohydrolases (Gal, Hex, β-Galactosylcerebrosidase, CatB and Cathepsin S (CatS)) were detectable also in peripheral districts such as blood plasma and the Peripheral Blood Mononuclear Cells (PBMCs) of AD patients, and that some of these alterations could discriminate AD from MCI [232,233]. In addition, gangliosides, which are substrates for some lysosomal enzymes [234,235], are altered in AD: monosialo-gangliosides are reported to increase in AD, while more complex gangliosides tend to decrease [202]. It was proposed that APP pathological processing could contribute to gangliosides alteration as AICD appears to down-regulate ganglioside GD3-synthase [236]. Moreover, GM1 and GM3 gangliosides are both capable of binding Aβ-peptide, and these interactions appear to occur early in disease progression, as they have been detected in brains that showed only the earliest signs of AD [202,236,237]. Other evidence of the involvement of gangliosides in AD pathophysiology came from the observation that the inhibition of glycosylceramide synthase, which catalyzes the first step in glycosphingolipid biosynthesis, was correlated with a reduction of amyloidogenic processing of APP [238].

Of note, AD patients showed different exosome profiles compared to MCI patients [239] and differences in synaptic proteins and autolysosomal markers (including CatD and Heat Shock Protein 70) have been found in AD patients’ exosome before symptoms onset [240,241], suggesting the potential use of exosomes as diagnostic biomarkers [212].

#### Potential Lysosomal Biomarkers for AD Diagnosis 

**Figure 6:**
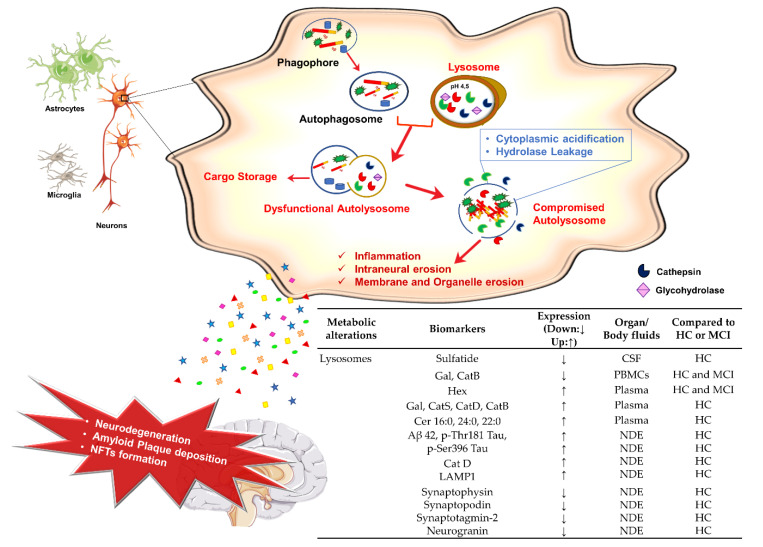
Lysosomes dysfunction and AD. Cartoon schematizes the main altered event leading to the alteration of lysosomal compartment and the table reports potential useful AD biomarkers as described in the text. The arrow direction (up ↑, down ↓) indicates the higher and lower expression levels of the related biomarker in AD with respect to healthy controls (HC) or MCI (Mild Cognitive Impairment). Aβ, β-Amyloid; CatB, Cathepsin B; CatD, Cathepsin D; CatS, Cathepsin S; Cer, Ceramide; Gal, β-Galactosidase; Hex, β-Hexosaminidase; LAMP1, Lysosomal Associated Membrane Protein 1; NDE, Neural-derived exosomes; NFTs, Tau Neurofibrillary Tangles.

The increasing evidence demonstrating the involvement of lysosomal system alterations in AD pathogenesis suggest that monitoring levels of the autophagy proteins as well as lysosomal enzymes and their products may be used as a biomarker for early diagnostic purposes (Figure 6).

For instance, the correlation of AD and Lysosomal Storage Disorders (LSDs) (a group of inherited diseases caused by mutations in lysosomal enzymes [242,243]) was confirmed by the detection of altered sphingolipid metabolism-related molecules in the CSF and serum of AD patients, thereby highlighting their biological relevance as possible biomarkers of the lysosomal proteins [244].

High baseline plasma levels of Ceramide (Cer) 16:0 and Cer 24:0 correlated with an increased risk of AD in older women, and increased Cer 22:0 and Cer 24:0 levels suggested hippocampal volume loss and cognitive decline [125]. Moreover, although a major decrease of CSF sulfatide was observed in the early stages of AD, very little change in its concentration was observed at the advanced stages [125,245,246] (Figure 6).

The levels of lysosomal enzyme Hex in plasma, Gal, and CatB in PBMCs from AD patients allowed discriminating AD versus healthy subjects and AD versus MCI [232,233].

Increased levels of lysosomal proteins (i.e., CatD and Lysosomal-Associated Membrane Protein 1, LAMP1) and decreased levels of synaptic proteins (synaptophysin, synaptopodin, synaptotagmin-2, and neurogranin) were also observed in the neural-derived plasma exosomes of AD patients [247] (Figure 6). Finally, neural-derived exosomes have been proposed as peripheral AD biomarkers. These exosomes, isolated from AD patients, showed significantly higher levels of Aβ1-42-peptide, Tau, p-Thr181 Tau, and p-Ser396 Tau (compared to controls), which provided a high predictability of disease development in the preclinical stage [247]. 

### 3.5. Metabolic Syndrome

In addition to the above-described metabolic alterations, metabolic syndrome also includes systemic organ dysfunction, hyperglycemia, insulin resistance, hypertriglyceridemia, low High-Density Lipoprotein (HDL) cholesterol, obesity, and hypertension [248,249,250,251,252]. As there is a strict correlation with systemic dysfunction, the identification of specific biomolecules as AD biomarkers is still under evaluation. However, FDG-PET imaging has evidenced that a gradual decrease in the cerebral glucose metabolic rate could discriminate patients with MCI that will develop AD from those who will not, suggesting that metabolic dysfunction may have a key role in the early mechanisms of AD [253].

A lipidomic study in the post-mortem human brain highlighted 34 metabolites that might differentiate AD patients from healthy controls [254]. These metabolites belong to pathways of some amino acids (alanine, aspartate, glutamate; arginine, proline, cysteine, methionine, glycine, serine, and threonine), purine metabolism, pantothenate, and coenzyme A biosynthesis [254], which were all altered in AD. This was also confirmed by metabolomic analysis of human plasma indicating the alterations of polyamine and arginine metabolism in MCI patients who afterwards developed AD [255].

## 4. Cross-Talk between Metabolic Dysfunctions, Neuroinflammation, and Neurodegeneration in AD

**Figure 7:**
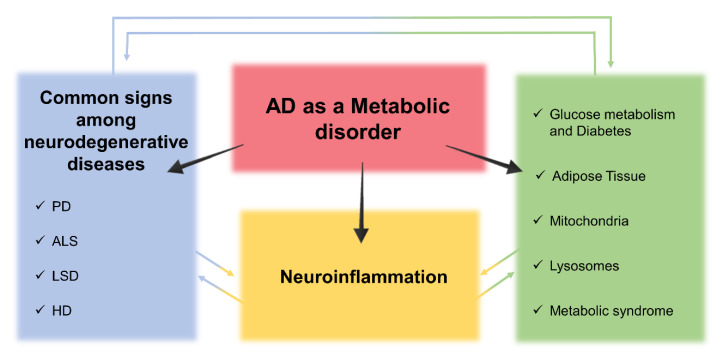
Cross-talk between metabolic dysfunctions, neuroinflammation, and neurodegeneration in AD. Schematic representation. AD, Alzheimer’s Disease; ALS, Amyotrophic Lateral Sclerosis; HD, Huntington’s disease; LSD, Lysosomal Storage Disorder; PD, Parkinson’s Disease.

The altered metabolic pathways contribute to the chronic status of neuroinflammation and neurodegeneration present in AD patients. In the next paragraphs, we highlight this cross-talk (Figure 7).

### 4.1. Neuroinflammation, Metabolic Alteration, and AD

The metabolic alterations occurring in AD described in the previous paragraphs all contribute to the worsening of the neuroinflammation state that characterizes this disease [256,257]. In AD, impaired glucose metabolism and insulin resistance are deeply correlated to the chronic inflammation state [79] as well as to adipose tissue that releases numerous pro-inflammatory adipokines [104,105,106,110]. Moreover, mitochondria dysfunctions promote oxidative stress and energy metabolism impairment [144,146], while lysosomal alterations lead to an impairment of the autophagic pathway and of its general degradation activity [197,200,212]. All the above events partake in the progress of AD neuroinflammation state [197,200,212].

Indeed, the CNS microglia and astrocytes have a central role in the neuroinflammation process occurring in AD [258,259,260,261]. In particular, the microglia has a phagocytic role that helps clear damaged neurons and remove pathogens other than facilitating tissue repair [262], while astrocytes are involved in many functions such as neurotransmitter uptake and recycling, the modulation of synaptic activity, and maintenance of the correct permeability of the BBB [260,261,263]. The integrity of the BBB in AD is compromised, and it is likely that both Tau and Aβ peptide may be involved in the loss of BBB integrity, exacerbating the neurodegenerative process and associated inflammatory responses [264]. In addition, further loosening of the tight junctions may be caused by the excessive release by the microglia of pro-inflammatory cytokines observed in AD, such as TNFα, IL-1β, and IL-17A [265,266]. Microglia appears to directly interact with soluble Aβ oligomers and Aβ fibrils via specific receptors including class A scavenger receptor A1, Cluster of differentiation (CD) 36, CD14, α6β1 integrin, CD47, and Toll-like receptors (TLR2, TLR4, TLR6, and TLR9) [267,268]. This binding leads to microglia activation that results in pro-inflammatory cytokines and chemokines production [267,268]. Moreover, astrocytes are highly activated in AD in response to excess Aβ peptide, which also leads to the upregulation of pro-inflammatory factors [269,270]. Among the different transcription factors, NF-κB is considered a primary regulator of inflammatory responses because its activation enhances BACE1 expression, thus stimulating the cleavage of APP and Aβ-peptide production [270]. In turn, BACE1 promotes the activity of secretases that enhance the production of Aβ peptide from APP. In detail, the reactive astrocytes express BACE and PSEN1 genes [270]. 

Finally, in a recent work, Ising et al. [271] have demonstrated that the inhibition of the NLRP3 (see the Abbreviation List) inflammasome activity in mouse models significantly reduced Tau phosphorylation in different brain regions and prevented cognitive decline, thus indicating that the NLRP3 inflammasome may have an important role in AD pathophysiology [271]. 

#### Marker of Neuroinflammation for AD Diagnosis

The molecules that partake in AD-related neuroinflammation processes are extensively studied as potential markers that could be monitored for early AD diagnosis. For example, a recent meta-analysis reported high CSF concentration of soluble TREM2, MCP-1, Chitinase 3-like 1 (YKL-40), and TGF-β in AD patients compared to healthy controls and increased tumor necrosis factor receptor 1 and 2 in peripheral blood of AD patients but not MCI patients [272]. 

TREM2 is a membrane receptor that plays a key role in mediating the phagocytic clearance during apoptosis, including the microglial phagocytosis of apoptotic neurons, and in modulating the inflammatory response caused by damaged myelin and amyloid plaques [273,274,275]. The tailored proteolytic cleavage of TREM2 at the H157–S158 peptide bond by A Disintegrin and metalloproteinase domain-containing protein (ADAM) 10 and ADAM17 proteases produces the soluble TREM2 [276,277], which generally is highly released in the CSF, where its levels are considered positive markers for neuronal injury [278,279]. For instance, the levels of soluble TREM2 increased in patients with autosomal dominant AD, and this increase was measured in the CSF of 218 subjects, 127 mutation carriers (MCs) and 91 noncarriers (NCs). In particular, the CSF levels of soluble TREM2 increased in MCs with respect to NCs 5 years before the appearance of symptoms, and these levels remained significantly higher until 5 years after symptoms onset [280].

In addition, peripheral cytokines levels such as IL-1β, IL-2, IL-6, IL-12, IL-18, IL-23, and TGF-β were seen to be altered in AD patients and could be used as candidate biomarkers [281,282,283,284].

However, when considering using peripheral inflammatory biomarkers for detecting asymptomatic phases of AD, it has to be taken into account that the alteration of these molecules is common to diverse neurodegenerative disease; thus, it is necessary to correlate their levels with other markers [282,285,286]. Nevertheless, these markers together with other characteristics of AD could serve as a peripheral panel that is useful to distinguish early AD from other neurodegenerative diseases.

### 4.2. Neurodegeneration, Metabolic Alteration, and AD

The numerous altered biological systems described so far in AD highlight the concept that the characterization of this neurodegenerative disease requires a deep understanding of many diverse pathological events. In this regard, it is crucial to underline that AD shares different pathological aspects with other neurodegenerative diseases, which could make early diagnosis difficult [287]. As a matter of fact, AD shares several features with Parkinson’s Disease (PD), Huntington’s disease (HD), and Amyotrophic Lateral Sclerosis (ALS) [286,287,288,289,290,291,292]. These common general pathways involve protein misfolding and aggregation, mitochondrial dysfunctions, oxidative stress and ROS production, neuroinflammation and phosphorylation impairment, and microRNA-altered expression [290,293,294], which appear to be changed concurrently [287,295,296,297,298,299]. 

The toxicity of misfolded and aggregated proteins is well-established, as it was observed in different neurodegenerative diseases (i.e., Aβ peptide in AD, α-Synuclein in PD, and Huntingtin in HD), and even though it occurs in different brain regions for each disease, confirms a crucial role of the toxicity of macromolecules accumulation in these disorders [300]. In this context, LSDs, due to their improper accumulation of disease-specific metabolites, share neural cell death, neurodegeneration, and other common interplay with the above-mentioned neurodegenerative diseases [287,301,302]. For example, cholesterol accumulates in late endosomes and lysosomes in the juvenile form of Niemann Pick type C disease characterized by progressive neurodegeneration similar to AD, including NFTs formation and increased APP amyloidogenic processing [206,303,304]. An extensive study reported APP-CTFs, Aβ peptide, and α-Synuclein accumulation in the Sandhoff mouse model [305,306,307] and ganglioside-bound Aβ-peptide in post-mortem human brains of patients with GM1 gangliosidosis and GM2 gangliosidosis [308]. Other evidence came from the presence of soluble Aβ(1-40)-peptide in post-mortem brains of patients with Mucopolysaccharidosis type 1 [309], in the mouse models of Mucopolysaccharidosis type IIIB [310], and the neuropathic form of Gaucher disease [311]. The accumulation of APP and APP-CTFs in the hippocampus of Neu1-deficient mice, a model of the LSD sialidosis, has also been demonstrated [202,211]. Common signs among AD, PD, HD, and ALS came also from the pathways regulating the clearance of misfolded and abnormal proteins that are associated with neurodegenerative diseases [312,313,314,315,316].

The toxic accumulation of proteins and metabolites is also partially responsible for the neuroinflammation process that is common to almost all neurodegenerative diseases. Today, innate immunity, with the particular relevance of glial cells, is considered to have a central role in brain homeostasis neurodegenerative events [317]. Indeed, sequencing and GWAS studies contributed to highlight the role of immunity and microglia as important contributors to the pathological events occurring in neurodegenerative diseases. This strengthened the idea of reversing degenerative events by reducing inflammation, even though it is still not clear at which time point this approach could be determinant [318].

Another common feature in neurodegenerative diseases is the oxidative stress caused by ROS and Reactive Nitrogen Species (RNS) brain accumulation [319,320,321,322]. The oxidative damage of different molecules within neurons (such as lipids, DNA, and proteins) was observed in AD, PD, HD, and ALS. In particular, ROS derived from Nicotinamide Adenine Dinucleotide Phosphate Oxidase 2 [320], which were detected in endothelial cells, platelets, neurons, astrocytes, and microglia, were suggested to be critical in mediating inflammation and apoptotic pathways in the CNS [261,321,323].

#### Neurodegeneration and Biomarkers for AD

The common pathways involved in neurodegenerative diseases, such as protein aggregation, neuroinflammation, and dysfunction in the metabolic and autophagic system, make the diagnosis of each specific disease more difficult. Currently, it is possible to clearly discriminate one neurodegenerative disease among others by combining several common and genetic-specific markers, whether possible. These include AD rare mutations in APP, PSEN1, and PSEN2 genes [324]; PD-specific genetic markers (e.g., in SNCA, LRRK2, PRKN, UCHL1, PINK1, DJ-1, NR4A, see Abbreviation List) [324]; ALS genetic mutations in more than a dozen genes that have been found to cause familial ALS (ALS2, NEFH, FUS, TARDBP, C9orf72, and SOD1 [325], see Abbreviation List); and the HD characteristic mutation in the Huntingtin gene [324]. Finally, the LSDs can be identified by disease-specific genetic mutations that lead to enzyme deficiencies within the lysosomes, resulting in an accumulation of undegraded substrate [326]. 

## 5. Concluding Remarks

In this review, we have documented the involvement of metabolic alterations in AD pathogenesis and highlighted the contribution of these findings to improving a suitable biomarkers’ panel for the early diagnosis of AD. Indeed, the field is under constant update due to the metanalysis investigations that integrate old and new findings from proteomic, metabolomic, and transcriptomic studies comparing AD versus healthy subjects or AD versus MCI or other neurodegenerative disorders [327,328,329]. Advancing is also from an available innovative in vitro cell model of AD or other neurogenerative diseases [330,331,332,333,334,335].

The overall findings provide a portfolio of biomolecules with potential therapeutic AD activity. This may help overcoming the absence of an effective therapeutic strategy for AD, as the available therapeutic agents are up to now just symptomatic treatments focused on improving cognitive symptoms: 3 acetylocholinesterase inhibitors (donepezil, galantamine, and rivastigmine) that help maintain high acetylcholine levels, and the noncompetitive *n*-Methyl-D-Aspartate (NMDA) receptor antagonist memantine used for counteracting glutamate excitatory neurotoxicity [336,337,338]. 

However, due to the discovery of the new AD biomarkers, there are currently 136 active trials involving 121 therapeutic agents at different stages. The disease-modifying drugs that have been studied in the last years that eventually reached phase 3 are mainly focused on counteracting (i) the deposition of extracellular amyloid β plaques mainly consisting of immunotherapy approaches and (ii) the synaptic plasticity or neuroprotection small molecules [339].

Small molecules in phase 3 are targeted to (i) Synaptic plasticity/Neuroprotection: AGB101, ANAVEX2-73, BHV4157, Icosapent Ethyl; (ii) Inflammation/Infection/Immunity: ALZT-OP1a, Azeliragon, COR388, Masitinib; (iii) Metabolism and bioenergetics: Metformin, Tricaprilin; (iv) Vasculature: Losartan+Amlodipine+Atorvastan and (v) Tau: TRx0237 [339]. In particular, TRx0237 (LMTX), which is a Tau aggregation inhibitor that decreases the level of aggregated Tau proteins [340], is a promising agent, and the ongoing phase 3 trial should be completed in 2022 [341]. Of note, 18F-FDG-PET is used as an outcome measure to monitor the efficacy of the treatment [341,342].

Other than small molecules, important immunotherapy strategies have been developed and represent another encouraging approach that may lead to the finding of some effective treatment for AD. Monoclonal antibodies have been developed to target Aβ and Tau oligomers, and the immunotherapy agents already in phase 3 are all targeted to amyloid: Aducanumab (Monoclonal antibody directed at plaques and oligomers), BAN2401 (Monoclonal antibody directed at protofibrils), Gantenerumab (Monoclonal antibody directed at plaques and oligomers), and Solanezumab (Monoclonal antibody directed at monomers) [339].

The active vaccination agent CAD106 (Amyloid Vaccine consisting of multiple copies of the Aβ1–6 peptide coupled to a carrier containing bacteriophage Qβ coat proteins) was a promising therapy that entered phase 2/3 in 2015 [343] and continued phase 3 in 2019, but in September 2019, Novartis noted that the CAD106 project had been retired [344,345].

Finally, the recent findings on the regulatory role of microRNAs in AD pathophysiology encouraged the possibility of modulating mRNA transcription using Antisense Oligonucleotides (ASOs). For example, ASOs targeting GSK-3β in Senescence-Accelerated Prone mice P8 mice improved memory and learning and decreased oxidative stress [346], while ASOs targeting human Tau expressed in one AD mouse model were able to ameliorate pathological Tau deposition [347].

In conclusion, the overall findings here reviewed envisage the great molecular complexity of AD pathogenesis, validate the difficulty to establish an early decision diagnosis, and also indicate a new roadmap that collects canonical AD hallmarks and new biomolecules coming from altered metabolic pathways.

## Figures and Tables

**Table 1:** The dominant inherited Alzheimer’s disease (AD) genes Amyloid Precursor Protein (APP), Presenilin 1 (PSEN1), and Presenilin 2 (PSEN2).

Gene	Chr.	Protein Length	Protein Domains	N° of Mutations			Reference
				Pathogenic	Non Pathogenic	Protective	
APP	21q21.3	770 aa	Extracellular	10	15	1	Alzaforum Database [13]
			Transmembrane	16	-	-	
			Intracellular	1	-	-	
PSEN1	14q24.2	467 aa	Extracellular	16	1	1	Alzaforum Database [13]
			9 Transmembrane	226	-	-	
			Intracellular	30	-	-	
PSEN2	1q42.13	448 aa	Extracellular	4	-	-	Alzaforum Database [13]
			9 Transmembrane	7	5	-	
			Intracellular	3	5	-	

**Table 2:** Genetic variant, max magnitude, chromosome position, and clinical characteristics of definite AD cases carrying risk variants of principal sporadic form of Alzheimer’s disease (SAD)-associated genes. ApoE: Apolipoprotein E.

Gene	Variant	Max Magnitude	Chr. Position	Clinical Features
TOMM40	rs10524523	3	44,899,792	Higher risk for late-onset Alzheimer’s disease
	rs157582	2.1	44,892,962	Weaker memory performance
	rs2075650	2	44,892,362	Possibly 2–4× higher Alzheimer’s risk
APOE	rs199768005	2.1	44,909,057	Marked reduced risk of Alzheimer’s disease
	rs429358	3	44,908,684	>3× increased risk for Alzheimer’s
	rs449647	2	44,905,307	Lower levels of ApoE
TREM2	rs104894002	6	41,161,557	Alzheimer’s, late-onset, possible/predicted
	rs143332484	2	41,161,469	Moderate increase (1.7×) in risk for Alzheimer’s disease
	rs75932628	3.5	41,161,514	Risk of Alzheimer’s disease
ABCA7	rs113809142	3	1,056,245	≈2× higher risk for Alzheimer’s disease
	rs115550680	2.5	1,050,421	Increased risk (≈2.2×) of Alzheimer’s, observed for African-Americans
	rs200538373	3	1,061,893	≈3x higher risk for Alzheimer’s disease
	rs72973581	2.5	1,043,104	Slightly lower risk (0.57×) for Alzheimer’s, according to one study
	rs78117248	2	1,052,854	Risk factor for Alzheimer disease (odds ratio ≈2×)
CLU	rs11136000	1.5	27,607,002	0.84× decreased risk for Alzheimer’s disease
CR1	rs6656401	1.5	207,518,704	1.18× increased risk for late-onset Alzheimer’s
	rs3818361	1.2	207,611,623	1.2× increased risk for late-onset Alzheimer’s
CD33	rs3865444	1.6	51,224,706	Slight reduction in risk for Alzheimer’s disease
MS4A6A	rs610932	1.5	60,171,834	An allele associated with reduced risk of Alzheimer’s in East Asian populations
BIN1	rs6733839	NA	127,135,234	This SNP has a population attributable fraction for AD of 8.1 which is second only to APOE4’s of 27.3
PICALM	rs3851179	1.5	86,157,598	0.85× decreased risk for Alzheimer’s disease
SORL1	rs10892759	1.01	121,593,379	Reduced risk for Alzheimer’s
	rs1784931	1.01	121,612,229	Reduced risk for Alzheimer’s
PLD3	rs145999145	2	40,371,688	2× higher risk for Alzheimer’s disease
CTNNA3	rs2306402	2.1	67,175,727	1.2× increased risk for late-onset Alzheimer’s disease
DNMBP	rs3740057	NA	99,898,828	Increased risk for late-onset Alzheimer’s disease in both Japanese and Belgian populations
	rs10883421	NA	99,912,584	Increased risk for late-onset Alzheimer’s disease in both Japanese and Belgian populations
BACE1	rs638405	2	117,293,108	2× increased Alzheimer’s risk in ApoE4 carriers
	rs4938369	NA	117,317,404	1.6× increased risk for Alzheimer’s
GAB2	rs7101429	2	78,281,921	0.70× reduced risk for Alzheimer’s risk
ADAM10	rs145518263	4	58,665,141	Rare mutation increasing risk for late-onset Alzheimer’s disease
	rs61751103	4	58,665,172	Rare mutation increasing risk for late-onset Alzheimer’s disease
ATP8B4	rs10519262	NA	50,140,297	1.9× risk for AD
ABCA2	rs908832	NA	137,018,032	3.8× increased risk for early-onset Alzheimer’s
OLR1	rs1050283	NA	10,159,690	Increased risk for Alzheimer’s
A2M	rs669	NA	9,079,672	3.8× or higher increased risk for Alzheimer’s
OTC	rs5963409	NA	38,351,716	1.19× increased risk for Alzheimer’s disease

Table 2 summarizes the main risk genetic variants correlated with AD. Only the entry with reported clinical futures are selected from the SNPedia database [26]. The full gene name is reported in the Abbreviation list. SNP: Single Nucleotide Polymorphism; NA: Not Assigned.

## Abbreviations

A2M	Alpha-2-Macroglobulin
ABCA2	Adenosine Triphosphate Binding Cassette Subfamily A Member 2
ABCA7	Adenosine Triphosphate Binding Cassette Subfamily A Member 7
ADAM10	A Disintegrin And Metalloprotease Domain 10
AK2	Adenylate Kinase 2
ALS2	Alsin Rho Guanine Nucleotide Exchange Factor Amyotrophic Lateral Sclerosis 2 APOE
ATP8B4	Adenosine Triphosphatase Phospholipid Transporting 8B4
BACE1	Beta-Secretase 1
BIN1	Bridging Integrator-1
C9orf72	C9orf72-SMCR8 complex subunit
CD33	Sialic Acid-Binding Ig-Like Lectin 3
CLU	Clusterin
CR1	Complement C3b/C4b receptor 1 (Knops blood group)
CTNNA3	Catenin Alpha 3
CYCS	Cytochrome C
DJ-1	Parkinsonism-associated deglycase
DLD	Dihydrolipoyl dehydrogenase
DNMBP	Dynamin Binding Protein
FUS	Fusion RNA binding protein
GAB2	Growth Factor Receptor Bound Protein 2 Associated Binding Protein 2
GATM	Glycine Amidinotransferase
HSPA9	Stress-70 protein
LRRK2	Leucine-Rich Repeat Kinase2
MS4A6A	Membrane Spanning 4-Domains A6A
NEFH	Neurofilament Heavy
NLRP3	Nucleotide-Binding Oligomerization Domain, Leucine-Rich Repeat and Pyrin Domain Containing 3
NR4A2	Nuclear Receptor Subfamily 4 Group A Member 2
OLR1	Oxidized Low Density Lipoprotein Receptor 1
OTC	Ornithine Carbamoyltransferase
PCK2	Phosphoenolpyruvate Carboxykinase 2
PICALM	Phosphatidylinositol Binding Clathrin Assembly Protein
PINK1	Phosphatase and Tensin Homolog-induced Kinase 1
PLD3	Phospholipase D family member 3
PRKN	Parkin RBR E3 ubiquitin protein ligase
SNCA	Synuclein alpha
SOD1	Superoxide Dismutase 1
SORL1	Sortilin Related Receptor 1
TARDBP	TAR DNA Binding Protein
TOMM40	Translocase of Outer Mitochondrial Membrane 40
TREM2	Triggering Receptor Expressed on Myeloid Cells 2
UCHL1	Ubiquitin C-Terminal Hydrolase L1
